# Type 2 Diabetes Mellitus Associated with Obesity (Diabesity). The Central Role of Gut Microbiota and Its Translational Applications

**DOI:** 10.3390/nu12092749

**Published:** 2020-09-09

**Authors:** Miguel A. Ortega, Oscar Fraile-Martínez, Irene Naya, Natalio García-Honduvilla, Melchor Álvarez-Mon, Julia Buján, Ángel Asúnsolo, Basilio de la Torre

**Affiliations:** 1 Department of Medicine and Medical Specialities, Faculty of Medicine and Health Sciences, University of Alcalá, Alcalá de Henares, 28801 Madrid, Spain; oscarfra.7@hotmail.com (O.F.-M.); ire_mikito@hotmail.com (I.N.); natalio.garcia@uah.es (N.G.-H.); mademons@gmail.com (M.Á.-M.); mjulia.bujan@uah.es (J.B.); 2 Institute Ramón y Cajal for Health Research (IRYCIS), 28034 Madrid, Spain; 3 Tumor Registry, Pathological Anatomy Service, University Hospital Príncipe de Asturias, Alcalá de Henares, 28801 Madrid, Spain; 4 Networking Research Center on Hepatic and Digestive Diseases (CIBER-EHD), 28801 Madrid, Spain; 5 Immune System Diseases-Rheumatology, Oncology and Medicine Service, University Hospital Príncipe de Asturias, 28801 Madrid, Spain; 6 Department of Surgery, Medical and Social Sciences, Faculty of Medicine and Health Sciences, University of Alcalá, Alcalá de Henares, 28801 Madrid, Spain; angel.asunsolo@uah.es (Á.A.); bjtorre@gmail.com (B.d.l.T.); 7 Department of Epidemiology and Biostatistics, Graduate School of Public Health and Health Policy, The City University of New York, New York, NY 10027, USA; 8 Service of Traumatology of University Hospital Ramón y Cajal, 28034 Madrid, Spain

**Keywords:** obesity, type 2 diabetes mellitus, diabesity, gut microbiota, dysbiosis

## Abstract

Obesity is a condition of rising prevalence worldwide, with important socioeconomic implications, being considered as a growing public health concern. Frequently, obesity brings other complications in addition to itself—like Type 2 Diabetes Mellitus (T2DM)—sharing origin, risk factors and pathophysiological mechanisms. In this context, some authors have decided to include both conditions as a unique entity known as “diabesity”. In fact, understanding diabesity as a single disease is possible to maximise the benefits from therapies received in these patients. Gut microbiota plays a key role in individual’s health, and their alterations, either in its composition or derived products are related to a wide range of metabolic disorders like T2DM and obesity. The present work aims to collect the different changes reported in gut microbiota in patients with T2DM associated with obesity and their possible role in the onset, development, and establishment of the disease. Moreover, current research lines to modulate gut microbiota and the potential clinical translation derived from the knowledge of this system will also be reviewed, which may provide support for a better clinical management of such a complex condition.

## 1. The Global Challenge of Obesity

Obesity represents one of the greatest public health concerns worldwide, being considered as an important risk factor for the development of chronic or non-communicable diseases (NCD). In addition, its economic implications also pose a serious warning for sanitary authorities [1,2]. According to the last updated records from the World Health Organization (WHO), approximately 40% of people around the world are overweight, whereas 13% are obese. The prevalence of this condition has importantly increased over the years, in such a way as to almost triplicate from the 1970s, importantly affecting women [3]. It is estimated that, in the world more people are presently overweight than underweight, and this situation is not only reported in western societies but also in developing countries, due to the lower cost of the obesogenic products [4]. In Europe, it is expected that by 2025 up to 20% of the inhabitants could develop obesity, but a higher percentage cannot be discarded [5]. Similarly, in Spain, recent data sustain that one in two adults are presently overweight, and around the 15% of them are obese [6]. Childhood obesity also represents a global threat, even more worrying because of its association with an increasing morbidity and mortality from early ages [7,8]. Overall, these statistics show the impact of obesity nowadays, and the necessity of deepening understanding in this important condition.

Obesity includes a variety of individual, social, economic, psychological, commercial, and environmental factors which may be considered to fully understand this condition [9]. To start with, obesity can be defined as an excessive accumulation of adipose tissue prolonged in time. The location and the amount of stored fat are two key factors inversely related to the health and well-being of individuals, limiting their quality of life [10]. The main criterion used for the assessment of obesity is the body mass index (BMI), which is the result of the quotient of the weight (measured in kg) divided by the size (in m^2^). Thus, a value ≥25 kg/m^2^ is diagnosed as overweight, and a value higher than 30, as obesity. However, this system is not enough to explain why, for example, some patients with a higher BMI were related to a minor risk of mortality, a fact known as the obesity paradox [11]. Hence, it is important to consider other factors such as fat redistribution, metabolic status, cardiorespiratory fitness, age or ethnic factors for a more accurate stratification to gain further insights into this condition [12,13].

On the other hand, obesity as a disease may be perceived as a set of disabling and pathophysiological problems with inherent comorbidities, a result of the interaction of multiple obesogenic factors [14]. Genetic predisposition is a major determinant in the outbreak of obesity, and the origin may be understood from an evolutionary point of view, where the presence of “thrifty genes” could represent an adaptation of the hominids to the scarcity of resources during the different eras in their history [15]. Additionally, epigenetic regulation may play a key role in the susceptibility to obesity, especially during pregnancy and the availability of nutrients in this period. Likewise, early postnatal development and paternal lifestyle factors seem to also be crucial to switch on/off gene expression [16]. Thanks to the progress in genomic techniques, more than a hundred genes directly related with obesity have been described, such as those controlling appetite or even thermogenic modulation [17,18]. Physical inactivity, an excessive intake, dietary patterns, or alcohol consumption are the most representative examples of environmental risk factors in obesity [19]. The interaction of environmental factors and genes and its effect on epigenetic modulation will finally lead to obesity [20]. Moreover, a lack of education in nutrition, cultural or marketing influence, the low price of unhealthy products and even the existence of certain standards of beauty, relationships or a poor self-esteem, may also be involved in the origin and establishment of obesity in our culture [21,22,23].

In this context, different organisations have risen to regulate and control such an extended condition, like the European Association for the Study of Obesity (EASO). This entity remarks the need for a multidisciplinary approach to maximise the success in the treatment of these patients, their quality of life and their general well-being [24]. Public health measures should also consider these various factors involved in the development of obesity, including economic and procedures assessments, as well as a clear, detailed, and consistent language for a better clinical management in the general population [25]. In the same way, the continuous progression and increasing incidence of this condition denote the lack of success of these measures, thus elucidating the necessity for a greater implication by governments, which may create awareness of this concern. For instance, it would be interesting to promote lifestyle changes in the general population, encouraging higher consumption of fruits, non-starchy vegetables or nuts and reducing the consumption of red or processed meat and high sugar and sodium foods [26], or encouraging people to regularly practice some exercise by the creation of installations or green spaces. Television campaigns or advertisements warning of obesity could also have a significant impact to educate the general population concerning one of the greatest public health issues.

## 2. Type 2 Diabetes Mellitus and Its Connexion with Obesity

Obesity is often related with a wide range of complications, including cardiovascular disease, metabolic disorders like type 2 diabetes mellitus (T2DM), chronic obstructive pulmonary disease, arthritis, cancer, and even psychosocial conditions [14,27]. This is due to the excessive adipose tissue and fat redistribution in obese patients, which is directly implicated with hyperglycaemia, hyperlipidaemia, insulin resistance, endothelial dysfunction, and chronic inflammation [28]. T2DM, also known as non-insulin dependent diabetes, is a condition frequently found in obese patients, and some authors consider them as a unique entity termed as “diabesity” [29]. In fact it is known that up to an 85.2% of people with T2DM have a problem of being overweight or obese [30] and by 2025, more than 300 million of people will have T2DM associated with obesity [31] so, in the majority of cases it is not possible to understand these pathologies separately.

**Figure 1:**
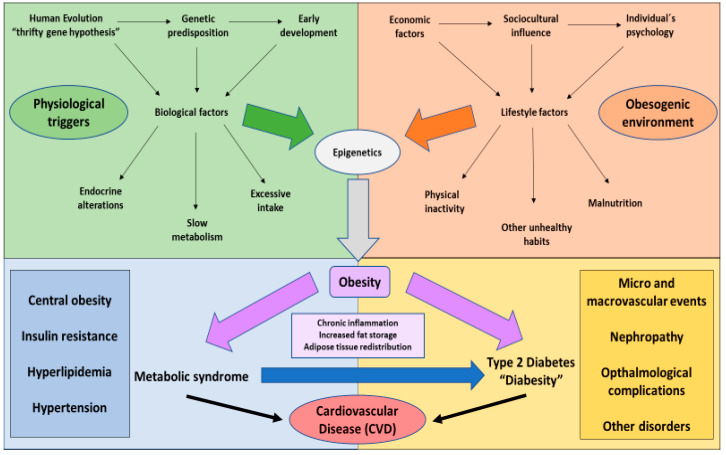
The interactions between multiple biological and lifestyle factors, directly influenced by an obesogenic environment and physiological changes may interplay with the individual’s epigenetic regulation, thus promoting the development of obesity. This condition characterised by an excessive fat storage, adipose tissue redistribution and chronic inflammation could lead to metabolic syndrome, a cluster of systemic alterations (central obesity, insulin resistance, hyperlipidemia and hypertension). The presence of metabolic syndrome increases the risk of developing type 2 diabetes mellitus (T2DM) in obese patients (Named as diabesity), as it has been reported that obese people without additional metabolic disorders rarely present T2DM. Diabesity is often related to T2DM complications like micro and macrovascular events, renal or ocular disease and other alterations, and importantly with cardiovascular diseases (CVD), a major problem of both diabesity and metabolic syndrome and the leading cause of death in obese patients.

Three hypotheses have been established to explain the relationship between these conditions: (1) chronic inflammation associated with obesity and their proinflammatory cytokines produced by macrophages in adipose tissue affects insulin dependent tissues and beta cells, (2) Lipotoxicity generated by the augmentation of ectopic lipid stores in obese people induce and promote the damage and cytotoxicity in peripheral tissues and (3) adipokines hypothesis which sustains that stressed adipocytes release a set of autocrine and paracrine products that finally conduct to the loss of insulin sensitivity and to the capacity of beta cells in the pancreas [32]. Equally, obesity is a condition that is associated to insulin resistance, which conducts to a chronic hyperglycaemia, thus causing T2DM. Moreover, other mechanisms have been described by which these conditions may be connected, such as the role of leptin. Leptin is a hormone responsible for controlling food intake thanks to its anorexigenic effect on the hypothalamus, having been observed how, in obese people, the levels of this hormone are increased, leading to a state of leptin resistance [33]. On the other hand, it has been reported that, consumption of hypercaloric foods and high fat diets is associated with a mitochondrial dysfunction and endoplasmic reticulum stress in the hypothalamus, thus promoting not only leptin but also insulin resistance [34]. Interestingly, it has also been demonstrated how this increase in leptin levels and a higher ratio with adiponectin are associated with an increase in the proinflammatory cytokines like TNF-α and IL-6, once again related to the insulin resistance and T2DM [35]. Similarly, it is known that from an evolutionary perspective, T2DM has appeared as a consequence of the imbalance of two key factors: “Metabolic capacity”—which promotes glucose homeostasis, and hence is antidiabetic—and “metabolic load”—the opposite to glucose regulation, being prodiabetic. Thus, dealing with this condition is intended to reduce the metabolic load, which in turn is enhanced by obesity or unhealthy lifestyles [36]. Frequently, the order of appearance is the following: As previously described, the exposure to genetic and environmental factors promotes the developmentof obesity. Obesity triggers and takes part in a complex cluster of conditions like central obesity, insulin resistance, hypertension, and hyperlipidemia, collectively known as metabolic syndrome or syndrome X [37]. The presence of metabolic syndrome is directly correlated with T2DM and other complications such as cardiovascular diseases (CVDs) [38]. In addition, T2DM is a major risk factor of micro and macrovascular events, nephropathies, opthalmological pathologies, cognitive and mood disorders, or bone metabolism impairments, amongst other complications [39]. The absence of metabolic syndrome reduces the risk of acquiring T2DM [40], thus denoting the complex interactions between obesity and multiple risk factors in the onset and establishment of T2DM, as represented in Figure 1.

## 3. Clinical Management of Diabesity

Because of the multiple links between T2DM and obesity, numerous studies have showed that by targeting obesity, it is possible to achieve a significant improvement in T2DM. Treating obesity consists, in a simple way, of reaching weight loss in these patients. Various strategies such as lifestyle interventions, pharmacotherapy, and even major procedures like bariatric surgery have been described [41]. Many studies have demonstrated not only how this clinical management is useful for losing weight, but also for ameliorating T2DM, notably improving their quality of life and life expectancies [42,43,44]. It is known that concomitant therapies like antidiabetics, antidepressants or antihypertensives may have important implications in weight gain and glycaemic profiles [41,45] and thus, denoting the potential effects of treating T2DM and obesity as a unique entity.

### 3.1. The Impact of Diet and Lifestyle Interventions in Diabesity

Diet and lifestyle interventions contribute significantly to clinical management of these conditions. The Mediterranean diet has proved to be one of the most effective choices in the clinical management of patients with diabesity and metabolic syndrome, to prevent CVDs complications [46] PREDIMED study (PREvención con DIetaMEDiterránea) represents accurately the impact of this intervention in people with obesity and T2DM, markedly in the prevention of their derived complications and global mortality [47,48]. In fact, thanks to interventional studies like this, it has been reported that implementing a Mediterranean diet rich in unsaturated fatty acids, is an interesting strategy with much greater efficacy than, for example, restricting the total fat intake in people with diabesity [49]. Likewise, other studies have found some common polymorphisms such as the transcription factor TCF7L2, which may be important in precision medicine as well as a potential predictive biomarker in patients with T2DM and obesity [50]. In addition, PREDIMED-Plus has also gained further insights into lifestyle interventions in an expanded way, including the monitoring of cognitive symptoms [51], quality of sleep and sleep patterns [52] and the importance of promoting and prescript moderate and moderate-to-vigorous physical activity for an improvement in inflammatory profiles and individual’s health [53].

On the other hand, several studies have also demonstrated the efficacy of other strategies in the nutritional management of diabesity. An interesting example of diet is the low carbohydrate (LC) diet. The basis of this diet, firtstly hypothesized by Atkins in the 1970s is that a high consumption of carbohydrates promotes the production of insulin, the primary cause of a higher intake and a slower metabolism. Therefore the LC diet will reduce insulin secretion, hence favouring weightloss [54,55]. Likewise, this strategy has also demonstrated its utility in patients with T2DM, improving the levels of blood glucose, glycosylated hemoglobin (HbA1c), as well as their lipid profile [56]. Following this line, Boden et al. [57] demonstrated the benefits of LC diet intervention for 2 weeks in a group of obese patients with T2DM, showing a reduction in their energy intake, weight loss, blood glucose levels, insulin sensitivity and other metabolic parameters. Equally, other studies have proved the positive effects of a very low carbohydrate ketogenic (LCK) diet in patients with diabesity. A randomized clinical trial conducted in 34 overweight adults with T2DM reported a significant reduction in weight loss and HbA1c, and received less medications than patients who received a moderate carbohydrate hypocaloric low-fat diet [58]. Notwithstanding, Johnston et al. [59] revealed that LCK and LC diets were similarly effective in reducing body weight and insulin resistance. Furthermore, the LCK diet was associated with adverse emotional and metabolic outcomes, warning about the dangers of this weight loss alternative. Additional dietary approaches such as the paleolitic diet or intermittent fasting seem to show promising effectiveness in patients with metabolic syndrome and T2DM, although further research is still needed [60,61]. Nevertheless, it seems that the diet, but also the adherence of each patient to the regimen, remains an essential point to successfully manage loss weight and glycaemic profiles of patients with diabesity. It is important to understand that is not all about the nutrients but also the origin and the processing of foods which may be considered when associating diet with health and disease [62].

### 3.2. Clinical Strategies in Diabesity Control

Bariatric surgery may pose an interesting alternative for the clinical management of obese patients with T2DM. An open label randomized clinical trial showed the potential benefits of bariatric surgery in a five-year follow-up cohort of patients with diabesity, but it required the continuous monitoring of blood glucose levels [63]. Nowadays, this procedure is indicated when the BMI > 35 kg/m^2^ (this value corresponds to a type II obesity) and it is necessary to be incorporated into a proper context of care and permanent medical assistance [64]. However, other studies demonstrated that this procedure may produce higher benefits in patients with a BMI ≥ 27 compared to intensive medical therapy in terms of glycaemia controlling and weight loss, reporting better results even in their quality of life [65], thus denoting the possibility of considering this alternative in the clinical management of diabesity. On the other hand, despite its effectivity, its high price, the possible complications associated, and the invasiveness of the procedure are other factors to be taken into account when selecting the proper therapy for each patient [66].

Pharmacotherapy is another field to consider in patients with T2DM associated with obesity. A suitable pharmacological treatment in diabesity should be able to promote weight loss and control blood glucose levels. According to the recommendations of the EASO, pharmacotherapy should be perceived as a part of the clinical management of diabesity but never an exclusive option, being specially recommended in patients with a BMI ≥ 30 kg/m2 or 27 kg/m2, in case of diabesity [67]. Furthermore, it is relevant to conduct an assessment after 3 months of therapy, classifying them by responders (>3% of weight less in patients with T2DM and obesity) or non-responders, considering different alternatives for the last group. Along this line, two major strategies may be targeted to achieve a favourable effect in diabesity: those aimed at weight loss with a positive impact in blood glucose profile and drugs specific to glycaemia control that reduce, or at least maintain the patients’ weight. [68]. The first therapies are mainly developed for controlling metabolism, satiety, and appetite [69]. For the second ones, some retrospective studies showed that the dual therapy with glucagon-like peptide 1 (GLP-1) agonists and sodium/glucose cotransporter 2 (SGLT-2) inhibitors alone or in combination with antidiabetic drugs could be especially useful in the treatment of diabesity [45].

Treating diabesity as a unique entity not only ameliorates T2DM and obesity, but also the establishment of related complications like high blood pressure [70]. It has been demonstrated that, the presence of these three conditions simultaneously reduce significatively the quality of life in these patients, regardless of sex [71]. In addition, it has been observed that the use of beta blocker agents in obese hypertensive patients could provoke weight gain, elucidating the necessity of looking for more effective alternatives [72]. Saxton et al. reported that, by targeting the excess of perivascular adipose tissue and its adipokines dysregulation, it is possible to obtain potential benefits in the clinical management of these pathologies [73], hence supporting the benefits of treating these alterations collectively rather than separately.

**Figure 2:**
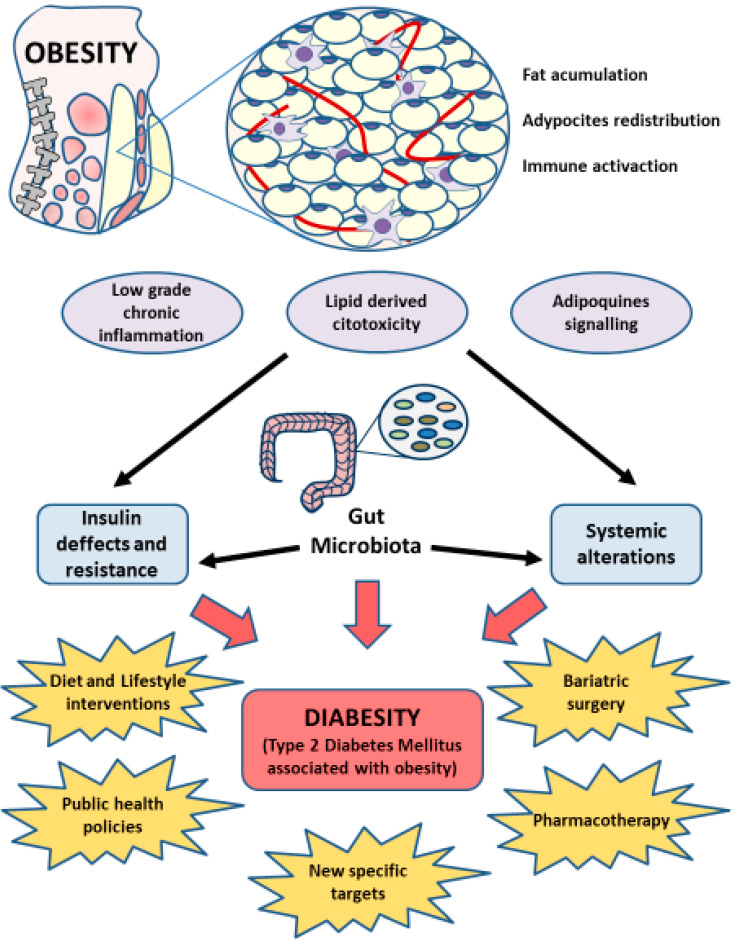
A general overview of diabesity. Lifestyle, sociocultural, genetic, and epigenetic factors promote the outbreak of obesity, characterized by an excessive accumulation of adipose tissue and fat redistribution. This status produces a low grade chronic inflammation, lipid dysregulation and its derived cytotoxicity, and a disruption in adipokines signaling which in turn provides a defective insulin production and sensitivity as well as a wide range of systemic effects, what finally will lead to diabesity (diabetes associated with obesity), with unique implications. Gut microbiota will also be affected in this condition, playing an important role in the development of diabesity, implicated with systemic and insulin alterations. Currently, the clinical management of diabesity is based on lifestyle interventions, public health policies or medical care like pharmacotherapy and bariatric surgery. However, it is important to continue searching for new specific targets, as each patient may present individual characteristics. Gut microbiota could represent an interesting alternative to achieve a higher precision and efficiency in the targeted therapy, hence denoting the importance of healthy habits which boost the prevention and awareness of this unique entity.

In this context, an increasing amount of evidence shows the potential role that gut microbiota may play in the development of T2DM and obesity, reporting the promising use of this target in the personalized and precision medicine [74]. Therefore, the main implications of gut microbiota in the origin and establishment of diabesity will be reviewed, as well as potential therapies that may benefit from the knowledge and understanding of this system (Figure 2).

## 4. Understanding the Role of Human Gut Microbiota

The term “human microbiota” refers to the totality of microorganisms inside the human body, both taking advantage of a bidirectional communication forming a single complex organism known as holobiont [75,76]. It is estimated that the amount of microorganisms in a human-being is around 3.8 × 10^13^, being found in a 10:1 proportion with eukaryotic cells, however some authors support that this relation is close to 1:1, with a total mass between 200 g to 1 kg of the total weight of a person [77,78,79]. On the other hand, microbiome refers to the whole microbial genome found in an individual, as well as their derived genic products, representing, approximately 100 times the human genome [80].

Although the microbiota is composed mainly of bacteria, the presence of a wide range of microorganisms, like archaea or viruses, has been reported (especially bacteriophages), and even more complex entities like fungi and even parasites—mainly protozoans and some helminths [81,82,83]. These microorganisms have coexisted and coevolved with human organisms, adapting themselves to accomplish certain functions, playing a key role in health maintenance [84,85]. These microbes can be inhabitant of a wide range of regions like the skin [86], the oral cavity [87], the upper airways and even in the genitourinary system [88,89]. However, it is in the gastrointestinal tract, and more specifically, in the gut, where the major microbiota is located, representing more than 70% of the total human microbiota, with multiple implications in various pathologies [90]. The importance of gut microbiota in human´s health and disease will be addressed in this section.

### 4.1. Composition, Diversity, and Dynamics of the Gut Microbiota

Many studies have attempted to study the “common” structure of human gut microbiota. Some projects like the Human Microbiome have pretended to elucidate this question to unravel which microbiome is the most typically associated either in health or disease conditions [91]. The gut ecosystem is represented by a core microbiome, composed of a microbiome present in all humans, or at least in the most part, in healthy individuals. In this line, Hugon et al. [92] isolated 2172 different species inside the gut, divided in 12 different phyla, observing how Proteobacteria, Actinobacteria, Firmicutes and Bacteroidetes constituted up to a 93.5% of the total microbes’ population. The group of Proteobacteria are mainly composed by the genus *Escherichia* and *Enterobacter*, whereas *Bifidobacterium* is predominant in Actinobacter phylum. Firmicutes are strongly represented by *Ruminiococcus*, *Clostridium* and *Lactobacillus* and *Bacteroides*, *Prevotella* and *Xylanibacter* are the major components of Bacteroidetes [93]. Among these, Firmicutes and Bacteroidetes are the most common phyla both in mice and humans and, although their proportion may change among individuals, their ratio seems to be important at different life stages [94] and also in the disease, as subsequently will be discussed. In addition, it has been reported the impact of other human gut microorganisms like Akkermansiamuciniphilla, the only member of Verrucomicrobia [95], *Methanobrevibactersmithii*, belonging to archaea, and fungi like Candida, whose proportion does not exceed the 1% [96]. Nevertheless, a variable part of the microbiome exists which may be characteristic of each person, according to the host phenotype, its physiological and pathophysiological status, lifestyle, their environment and even the moment, since some microbial communities cannot persist and colonise the gut [97]. In fact, it must be highlighted that there is such a huge microbial variability that each person could be said to present a distinctive microbial footprint. Moreover, it is known that microorganisms are distributed unequally throughout the gut, fluctuating from approximately 10^3^ cells in the duodenum to 10^12^ microbes at the descending colon [98].

Microbiota diversity largely depends on the habitat conditions. Some locations, like the skin, show higher variability in their communities compared to other regions as gut or oral cavity [99]. Development may also have a prominent role in gut microbiota composition. Initially, it was believed that the first microorganisms colonised human gut immediately postpartum. In contrast, currently it is thought that a maternal transference during pregnancy is produced, possibly through the placenta, umbilical cord, or amniotic liquid [100,101,102]. According to the form of labour, the initial newborn microbiome may be related to mother’s vaginal and faecal microbiota, if it is a natural birth, whereas if it is a caesarean one, it would be similar to the skin microbiota [103]. In the last group a reduction in microbial populations has been reported, resulting then in an initial alteration with a delayed Bacteroidetes colonisation and a minor efficiency of Th1 mediated responses for 2 years in comparison to those who were born by vaginal delivery [104]. On the other hand, other studies have shown that, nearly a month later this difference may disappear, by virtue of breastfeeding [105]. Lactation is one of the most important determinants of microbiota, due to the transmission of microorganisms from the mother, also containing antibodies or certain bioactive compounds like oligosaccharides, which make a major contribution in the microorganism–host interaction, the growth and the establishment of infant microbiota [106,107]. Curiously, differences in gut microbiota composition in babies fed with formula milk when compared to breastfeeding groups have been reported, denoting the impact of lactation in microbiota composition [108,109]. Afterwards, solid foods foster an increase in Bacteroidetes and Firmicutes, facilitating the digestion of carbohydrates, stimulating the diversity of the bacterial ecosystem, detrimental to Bifidobacterium, highly related to breastfeeding [110]. Interaction with objects and people may also have an impact during the first years of life [111]. Some studies also show how other factors like gestational age, host genetics or maternal diet could be equally important for the establishment of these first microbial communities [112]. At the age of three it is said that the gut microbiota is closely related to that of an adult. Despite this fact, external and internal factors of the individual make gut microbiota susceptible to change at any period of life [113]. Diet and lifestyle [114], BMI [115], hormones [116,117,118] and even country of residence and ethnicity [119] are some of the most relevant factors in adulthood to mention, also being elderly, associated with a loss of microbial diversity and the atrophy of intestinal mucosa, frequently aggravated by sedentarism and poor diets [120,121].

### 4.2. Main Functions of Gut Microbiota

The gut microbiota fulfils a variety of physiological functions which are crucial for host health, being an interesting subject of study from a preventive point of view [122]. The gut microbiota is implicated in a wide range of biological processes such as digestion of some nutrients and energy balance homeostasis. This is due to the production of proper bioactive metabolites, like short chain fatty acids (SCFA), which are butyrate, propionate, and acetate, considered as signalling molecules in gut and extraintestinal tissues [123]. The synthesis of SCFAs is obtained from the colonic fermentation of the named microbiota accessible carbohydrates (MAC)—the non-digestible polysaccharides present in the dietetic fibre [124]. Butyrate is principally produced by Firmicutes activity, whereas propionate and acetate are typically from Bacteroidetes [125].

Gut microbiota also contribute to the transport and metabolism of carbohydrates and amino acids like tryptophan, operating in the production of fat-soluble vitamins like vitamin K and water-soluble ones such as those belonging to B complex [126]. Additionally, the gut microbiota also participates in the conversion and metabolism of bile acids, as well as the biotransformation and elimination of xenobiotics and drugs [127].

In the same way, it could be said that the gut microbiota is strictly necessary to induce and educate the immune system, regulating in a local and systemic way, the activity of leukocytes [128]. Another point to outline is its decisive mission to maintain the integrity of the intestinal mucosal barrier, not to mention to prevent the colonisation by pathogens, mostly due to competition or to the production of antimicrobial substances [129]. Eventually, it has also been proved that the gut microbiota regulates neurological and psychological process, through the well-known microbiota–gut–brain axis [130,131]. Overall, it is undoubted that the gut microbiota and its numerous functions are vitally important for the individual´s health and studying its alterations would help to better the understanding of human diseases.

### 4.3. Gut Microbiota in Disease

In order to keep their functions, an adequate homeostasis in the composition, so-called eubiosis, is needed. This balance is disrupted under stress conditions like, for instance, lifestyle modifications, antibiotic misuse or changes in the immune system and intestinal mucosa, reducing microbial diversity and leading to a condition known as dysbiosis [132]. Furthermore, this dysbiosis situation may be exacerbated by an increase in oxidative stress, by the action of bacteriophages or the production of bacterial toxins, with all the consequences associated [133]. On the one hand, the production of many microbial compounds may be disturbed and this is often accompanied by an increment in gut permeability, which permits that some bacteria and their derivatives, like lipopolysaccharides (LPS), accessing to the bloodstream and producing, as a result, a status known as endotoxemia, with systemic involvements [134]. Moreover, some microorganisms in the gut microbiota are considered opportunistic pathogens, and may provoke adverse effects when dysbiosis occurs [135]. These are the reasons why human gut microbiota disorders are related to a broad range of intestinal pathologies, such as intestinal bowel disease, irritable bowel syndrome or celiac disease, as well as extraintestinal diseases like metabolic disorders, T2DM, obesity, cancer or nervous system affections, Alzheimer disease, Parkinson and even autism spectrum disorder [136]. Even so, it is complicated to establish if microbiota alterations are a cause, consequence or simply an adaptation to the pathological conditions of the individual [137,138]. Regardless, there are ever more studies contemplating the therapeutic validity of microbiota, in order to treat several complications [139,140], elucidating the importance of focusing our insights towards the study of gut microbiota, particularly a better understanding and management of health and disease.

## 5. Gut Microbiota in Diabesity

Changes in gut microbiota composition have been related to a wide variety of metabolic events such as an increase in adiposity, dyslipidaemia and T2DM. These disorders bring about an increased gut permeability, disrupting bile acid metabolism, serum levels of lipopolysaccharide and affecting SCFA production and function [141].

Firmicutes/Bacteroidetes ratio is a parameter that most importantly will be affected in these patients, having an increased Firmicutes community and decreased Bacteroidetes [142,143]. Furthermore, this ratio increases with factors like BMI [144] or fasting blood glucose levels [145]. Both phyla occupy different functional niches in the gut ecosystem. However, it is difficult to interpret this ratio now that, for example, Firmicutes has members like Clostridium botulinum, which may act as an opportunistic pathogen, or *Eubacteriumrectale*, *Roseburia* spp. and *Faecalibacteriumprausnitzii*, which are the main butyrate producing bacteria, and, in general terms, can be considered as beneficial for health [146,147]. Rising Firmicutes is usually associated to a poorer metabolic pattern, lower levels of glycan-degrading enzymes and an inverse relationship with resting energy expenditure [148,149]. A minor Bacteroidetes proportion, not only reduces microbial diversity, but also may affect energy metabolism since bacteria from this phylum are essential for providing energy to their host by propionate production, which may suppose 10% of daily calories when having a high fibre diet [150]. Additionally, this Bacteroidetes decrease implies a considerable reduction in acetate and propionate production, and, despite the major Firmicutes ratio, it has been found that there is a reduction in butyrate production, and its producing species, linked to diabesity [151].

Butyrate collaborates in lessening gut permeability, lessening appetite through the gut–brain axis which affects the vagus nerve, improving insulin sensitivity and energy metabolism, and it has been involved in fat oxidation, activating brown adipose tissue (BAT) [152,153]. Propionate arrives to the liver by portal circulation, acts on beta-pancreatic cells and influences the altered reward system in diabesity. Moreover, either propionate or butyrate, promote intestinal gluconeogenesis, affecting energy and glucose homeostasis [154]. Acetate is a SCFA which is released mainly to peripheral tissues and plays a crucial role in lessening appetite when linking to hypothalamic receptors [155,156]. It has been observed in obese animal models how there is a significant increase in faecal acetate concentration, promoting an increase in insulin and ghrelin secretion, provoking adipose tissue accumulation [157]. Nevertheless, other studies have shown that there was an increase in faecal acetate in healthy slim mice [158], so the role of acetate in metabolic disorders remains unclear. Even so, it is thought that along with butyrate, they can epigenetically regulate histone deacetylase enzymes, inhibiting them [159]. SCFAs also regulate the maintenance of the intestinal epithelial barrier, from the moment they act on G protein-coupled receptors (GPCR), promoting the release of the GLP-1, the inflammatory response and insulin sensitivity by adipocytes [160,161]. Hence, minor proportions of SCFA and their associated producing bacteria will be important in diabesity pathophysiology. In addition, it has been described how dysbiosis in obesity and T2DM promotes intestinal barrier disruption, which in turn supposes an entrance way to Gram-negative LPS, increased in the blood because of this fact, bringing a situation of metabolic endotoxemia [162,163]. Some animal models show endotoxemia as a crucial event in the beginning and development of diabesity [164]. Rising LPS levels cause chronic inflammation when joining to CD14/TLR4 receptors on macrophages, triggering a release of proinflammatory cytokines and leading to an increase in adipose tissue, hepatic insulin resistance and glucose intolerance [93]. Interestingly, Vérges et al. demonstrated that in T2DM patients, there was a stunting in LPS degradation, so the disease itself promotes or keeps associated endotoxemia, suggesting a potential therapeutic target for better clinical management in these patients [165]. Elevated levels of LPS in serum are also related to elevated concentrations of IL-6 and TNF-alpha in adipocytes [160]. Moreover, it is known that LPS, SCFA, tryptophan metabolites and other bacterial products are able to stimulate the nervous system directly by the vagus nerve or by immunological or neuroendocrine pathways, like leptin or insulin signalization [166,167]. The endocannabinoid system is now becoming more relevant for the gut–brain axis, also being important for energy and glucose metabolism. It is known that different products and bacterial communities may regulate this system, and at the same time may regulate bacterial communities due to their systemic distribution [168,169]. It has been proposed that endotoxemia might have a crucial implication in the hyperactivation of the endocannabinoid system in hypothalamus, with orexigenic effects or stimulating the intake, because of an increase in blood–brain barrier permeability which also induces neuroinflammation due to LPS effects on glia via TLR4 receptors [170]. In addition, it has been discovered that by interceding in these bacterial communities, it is possible to modulate the endocannabinoid system; as a remarkable example, *Akkermansiamuciniphilla* is a bacterium which is found to be decreased in diabesity, and it is considered to be notably important for keeping the intestinal integrity, and for lipid and glycemic metabolisms, and in the lessening of sustained endotoxemia as well [171]. Another point is that alterations have also been found in methanogenic archaea like *Methanobrevibactersmithii* [172]. *Prevotella* sp., *Bacteroides* sp., *Intestinibacter* sp., *Escherichia coli*, *Desulfovibrio* sp. or *Lactobacillus* sp., which seem to be important in this diabesity condition too, with diet being one of the most determinaning factors modulating microbiota in these patients [173].

**Figure 3:**
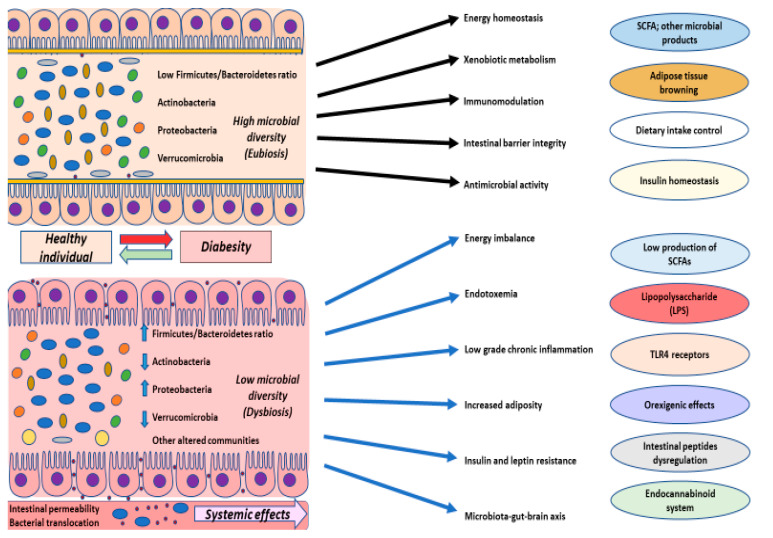
Changes described in gut microbiota of patients with diabetes-associated obesity. These patients show a minor wealth in microbial diversity, characterized by a high Firmicutes/Bacteroidetes ratio, that goes with modifications in Actinobacteria, Poteobacteria and in other microbial communities like methanogenic archaea or *Akkermansia muciniphilla*, the only Verrucomicrobia. These alterations may have important consequences in energy imbalance, endotoxemia, low-grade inflammation, higher adiposity, or insulin and leptin resistance, notably interacting with the microbiota–gut–brain axis. Different points of study have been examined to explain these changes. For instance, minor production of short chain fatty acids (SCFAs) and other microbial products, the presence of LPS in blood and its interaction with TLR4, associated with a systemic proinflammatory state, or the dysregulation in the production of intestinal peptides like glucagon-like peptide 1 (GLP-1) or glucose-dependent insulinotropic polypeptide (GIP), with orexigenic effects. The endocannabinoid system also plays an important role in the dysbiosis in patients with diabesity, who, moreover, show a lower ability for the browning of adipose tissue and insulin homeostasis.

To sum up, as shown in Figure 3, variations in gut microbiota composition, and its derived products are associated with an increase in adiposity, low-grade inflammation and insulin resistance, along with alterations in the endocannabinoid system, intestinal peptide production, leptin resistance and other metabolic characteristics associated with changes reported in patients with diabesity [174,175,176]. Therefore, targeting the microbiota and its related compounds, we may understand and maximize the results of existing therapies such as lifestyle interventions, and importantly diet, bariatric surgery and even received pharmacological treatment. Furthermore, it has been reported that focusing on microbiota also makes it possible to treat mood disorders derived from diabesity [177]. Hence, taking gut microbiota into consideration might consititute a very useful target for understanding this condition.

## 6. Importance of Gut Microbiota in Clinical Management of Diabesity

### 6.1. Diet and Lifestyle Interventions in Diabesity. Prebiotics and Probiotics

Diet is one of the main triggers of diabesity, also being a key modulator of gut microbiota composition [178,179,180]. For instance, the western dietary pattern, abundant in ultra-processed products, with sugar, fats, and other refined compounds and scarce in plant-based foods has been demonstrated to boost Firmicutes expansion, in particular Erysipelotrichia class, while reducing Bacillus and Bacteroidetes [181,182]. The use of some additives in this diet as artificial sweeteners has been associated with an increase in *Bacteroides* sp. but this could derivate rapidly in an impairment in glucose tolerance, followed by a decrease in *Lactobacillus reuteri* [183]. An excessive intake of saturated fatty acids was also related to diabetes progression in mice, having been related to a remarkable augmentation in bacterial translocation [184]. Furthermore, this diet promotes a higher production of chylomicrons, which in turn leads to a significative postprandial endotoxemia, due to their role in LPS transportation [185,186].

Many dietary interventions have been described to specifically modulate gut microbiota. Among them, some studies have shown that through a hypocaloric diet, low in fats or carbohydrates may promote an increase in Bacteroidetes as well as diminishing Firmicutes [187,188]. Notwithstanding, when combined with a Mediterranean diet this strategy maximises the results. The Mediterranean diet has been linked to a wide range of benefits in patients with diabesity, in part, due to their ability to regulate microbial populations, enhancing the growth of *Lactobacillus* sp., *Bifidobacterium* sp., y *Prevotella* sp., as well as limiting *Clostridium* sp. development [189]. This diet rich in fibre and some plants compounds was associated with higher levels of *Akkermansia muciniphila* and *Faecalibacterium prausnitzii*, providing a reduction in endotoxemia in patients with T2DM [190]. There are some specific foods which might be interesting to consider in clinical management of diabesity, as cocoa, or cinnamon, because of their high content of polyphenols, a pivotal modulator of gut microbiota. This compound has been implicated with positive effects in insulin sensitivity, glucose homeostasis and with other metabolic parameters [191,192]. The abundance of polyunsaturated fat in mediterranean diet, specially in eicosapentaenoic (EPA) and docosahexaenoic (DHA) fatty acids has been shown to promote Roseburia genus and *F. prausnitzii* populations, having a protective effect against T2DM [193]. The impact of other diets like gluten-free or ketogenic diet in gut microbiota remain to be elucidated [189,194].

**Figure 4:**
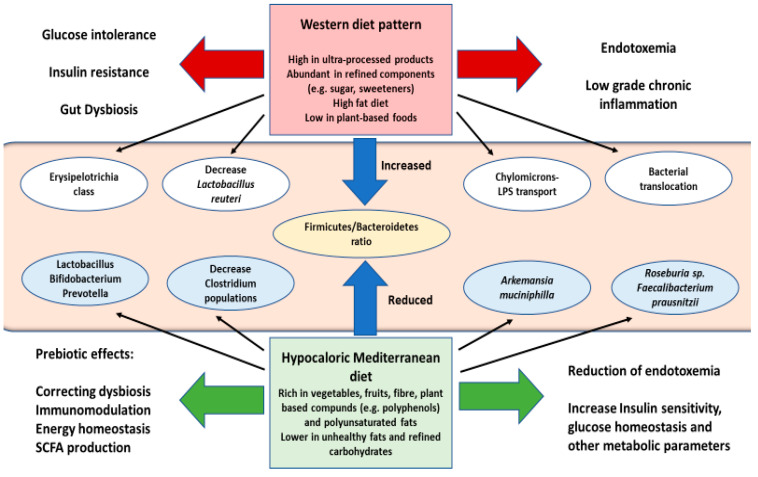
A representation of the various effects that diet may have in gut microbiota composition in patients with diabesity. Western dietary patterns, rich in ultraprocessed food, refined and unhealthy products could affect individual´s microbiota populations, promoting an increase in Firmicutes/Bacteroidetes ratio and gut dysbiosis, leading to a bacterial and LPS translocation, directly associated with endotoxemia and chronic inflammation, as well as the loss of glucose tolerance and insulin resistance. The Mediterranean diet is a powerful tool in the nutritional management of gut dysbiosis in patients with diabesity, mainly through their prebiotic effects, also related with the energy homeostasis, SCFA production and immunomodulation, along with a reduction in endotoxemia and promoting insulin sensitivity or glucose regulation.

The Mediterranean diet is one of the most interesting strategies when managing microbiota composition in patients with diabesity (Figure 4). The success of this diet in correcting gut dysbiosis is, mostly thanks to its prebiotic compounds [195]. Prebiotics are substances contained in certain foods which may modulate the composition of gut microbiota, thereby influencing energy homeostasis, satiety and weight control, SCFA production, supressing the growth of pathogens and immunomodulatory actions [196,197]. A great variety of prebiotics, such as inulin and fructooligosaccharides, galactooligosaccharides, polydextrose, lactulose, ciclodextrines, xilooligosaccharides or triphala has been decribed. The properties of these prebiotics, along with the foods or products in which they may be found, have been collected by Green et al. [198].

Probiotics may also make a major contribution to the clinical management of diabesity. Probiotics could be defined as products or foods containing living microorganisms, which in an adequate number may result beneficial to the host health [199]. Probiotics use different strains of Bifidobacterium and Lactobacillus having been associated with a reduction in BMI, diastolic pressure, blood triglycerides, and blood pressure [200,201]. The mechanisms by which probiotics assist these processes, are their potential to modulate gut microbiota, competing in the adhesion to intestinal mucosa and epithelium, encouraging mucus production and reinforcing the intestinal barrier [197]. The establishment of effective formulas and the search for the proper dose, duration, method of administration and long-term effects, are the major challenges for clinical translation of probiotics in diabesity [202]. Nonetheless, prebiotics and probiotics represent such a promising line of research in the clinical management of this condition. Recently, an interventional clinical trial in 41 patients with diabesity has been conducted to assess the use of prebiotics and probiotics in comparison to placebo group, studying some factors like gut microbiota composition, endotoxemia, neutrophils activation, beta cells function, gut permeability and quality of life in these patients (NCT02637115). Overall, these studies show how the use of prebiotics and probiotics may play an important role not only in treatment but also in prevention of obesity and T2DM in risk groups.

Finally, an appropriate rest and physical activity may also promote the establishment of a healthy gut microbiota [203,204]. Once again, it is important to harbour a multidisciplinary approach for the successful clinical management of such a complex disease.

### 6.2. Gut Microbiota and Bariatric Surgery

As previously outlined, bariatric surgery is a procedure which reports multiple benefits in weight loss, while improving glycaemic index in patients with diabesity. In fact, a 40–80% of obese patients who undergo this surgery also recover from their T2DM [205]. One cause explaining this outcome, is the regulatory role of bariatric surgery in gut microbiota, withits capacity to overcome gut dysbiosis having been demonstrated [206]. Moreover, it is known that bariatric surgery directly affects the production and enterohepatic circulation of bile acids [207]. The gut microbiota plays a key role in the biosynthesis and biotransformation of bile acids, which, in turn regulates the microbial populations through different signalling routes [208]. One study conducted by Zhang et al. [209] showed a significant reduction in Firmicutes and methanogenic archaea, while a rise in Gammaproteobacteria was found. Proteobacteria are stimulated by the presence of bile acids in the intestinal lumen, thus reducing the content of secondary bile acids while increasing primary bile acids [210]. Higher serum levels of these bile acids were associated with an enhanced metabolic and hormonal profile of the patient, possibly through their effects on the Farnesoid X Receptor (FXR) and G coupled protein receptor TGR5 [211].

Likewise, the study of microbiota and its metabolites are supposed to be a valuable point of study prior to bariatric surgery procedure. Ceperuelo-Mallafré et al. [212] found how lower levels of circulating basal succinate seemed to be useful to predict diabetes remission in 1 year. What is more, circulating succinate was still reduced up to a year after surgery. The role of succinate in obesity and metabolic disorders remains elusive. It is believed that in diabesity it may act as a marker of gut dysbiosis, acting as a signalling molecule in peripheral tissues, like SCFA [213]. Levels of succinate are correlated with an impaired glucose metabolism, being associated with an alteration in the ratio of Prevotellacea, Veillonellaceae, Odoribacteracea and Clostridacea [214].

Eventually, Murphy et al. [215] compared the effect of two types of bariatric surgery on the gut microbiota: Roux-en-Y gastric bypass (RYGB) and sleeve gastrectomy (SG). Interestingly, they reported more benefits in RYGB surgery, although they found that, regardless of the method performed, patientes who achieved the remission submitted an increase in Roseburia populations. In animal models, however, it has been shown that these procedures may modulate gut microbiota in a different way. While RYGB promote the growing of Proteobacteria/Gammaproteobacteria, SG held a major impact in Actinobacteria, with both surgeries resulting in a reduction in blood glucose [216]. On the other hand, a different study only replicated this outcome when RYGB was carried out [217]. These authors described an inverse relationship between Gammaproteobacteria population with postoperative body weight, resulting as an important variable to stabilize weight loss after undergoing bariatric surgery.

### 6.3. Gut Microbiota in Pharmacotherapy

Metformin is a first line therapy used for T2DM implicated in the reduction in hepatic gluconeogenesis and insulin production. Beyond these effects, some studies have endorsed its potential use in loss weight, mainly due to the impact of metformin on food intake centres and as a gut microbiota regulator [218]. More precisely, Sun et al. observed that after 3 days of metformin treatment, a significant decrease in the *Bacteroides fragilis* population was reported, followed by an increase in Glycoursodeoxycholic acid (GUDCA) [219]. This was associated with the inhibition mediated by FXR. Likewise, the positive actions of metformin in the higher production of SCFA, as well as in the growing of the bacteria *Akkemansia muciniphila* have been reported [220].

The role of antibiotics in diabesity have not been clarified yet. An abuse in the use of these substances is directly related not only with the generation of resistant microorganisms, but also with the promotion of some pathological conditions like obesity, as evidenced in numerous animal models. [221]. This is due to their impact on gut microbiota. Despite this, further studies are needed in humans. Rasmussen et al. showed that, recurrent exposition to antibiotics in childhood, importantly before the first 6 months increased the risk of becoming overweight and obese [222]. Similarly, Mikkelsen et al. demonstrated how the use of some antibiotics like narrow spectrum penicillin were related with a higher odds ratio of presenting T2DM [223], thereby concluding that over exposition to antibiotics may play an important role in the origin of diabesity. On the other hand, previous works have indicated that the use of some antibiotics or antitoxins could report substantial improvements in loss weight and insulin sensitivity in animal models with established diabesity [224], probably by their implications on gut dysbiosis.

The opening of new potential targets like incretins are proposed to be useful in the treatment of diabesity [225]. Incretins are hormones secreted by enteroendocrine cells which promote the secretion of insulin by beta cells. There are two types of incretins, glucose-dependent insulinotropic polypeptide (GIP) or glucagon like peptide 1 (GLP-1). Disruptions in the production and action of these products have been reported in a broad spectrum of pathologies, among them diabesity [226]. Equally these authors have proposed the existence of a complex interaction between nutrients, the gut microbiota, endocannabinoid system and enteroendocrine cells. Co and triagonist of the incretins-based therapies have shown promising results in preclinical studies to reduce body weight and blood glucose in patients with diabesity [225]. It is of note that, by targeting the microbiota and its interactions with this axis could act as a hopeful therapy for clinical management in patients with diabesity.

## 7. Conclusions and Future Perspectives

Obesity is a global threat defined by the interaction of numerous factors, entailing a high economic burden since being associated with many complications, like T2DM. Evidence collected supports the importance of treating both pathologies as a unique entity, thus maximising the benefits of the therapies received by these patients. Changes in gut microbiota are a common characteristic of a wide range of diseases, among them, diabesity. Gut dysbiosis and disruption of their microbial products may be involved in this condition, although more studies are needed to possibly establish causality in this relation [227]. In any case, it is undeniable that focusing on gut microbiota reports substantial improvements in the clinical management of diabesity, specially through diet. Moreover, it could be an important approach when considering other therapeutic options such as bariatric surgery or pharmacotherapy. Nevertheless, more lines of research are simultaneously opened, like the faecal microbiota transplant (FMT) or phage therapy.

FMT consists of changing the composition of gut microbiota by the extraction and purification of the faecal microbiota from a healthy patient and the introduction in a receptor via endoscopy or oral administration [228]. Currently, FMT is indicated in *Clostridium difficile* infections, but its use in other conditions has not been well-examined yet [229]. Current research aims to extend FMT in a wide range of intestinal and extraintestinal diseases in which gut dysbiosis is observed. A recent review showed that this method could be effective in the treatment of some patients with obesity and metabolic syndrome, although its therapeutic success depends on the receiver’s microbiome richness, as well as the metabolic status of the donor [230]. Conclusively, it is still necessary to accomplish further research to approve their use in patients with diabesity and the possible methods followed during this procedure, although the potential of these kind of techniques may represent an attractive therapeutic window in the future of diabesity.

Phage therapy is another strategy which is now being investigated to treat dysbiosis in several conditions [231]. Bacteriophages represent up to 90% of the viral populations composing gut microbiota, take a leading role regulating bacterial populations, also interacting with the immune system [232,233]. The origin of this therapy took place in the eastern Europe, almost a century ago. Further studies are needed to assess its efficacy and security though [234]. Rasmussen et al. have reported how transferring faecal bacteriophages from normal weight to obese mice promotes weight loss in the last group, besides an improvement in glucose parameters [235]. In addition, the use of phage-derived products poses an interesting area of research in the clinical management of diabesity [236]. The childhood could be an interesting period to target gut microbiota, either using probiotics, prebiotics, phages or a combination of these techniques [237], and clearly strengthening a proper lifestyle. However, currently, there are no clinical trials using phage therapy in patients with diabesity.

Browning is a novel treatment with such a potential in the treatment of diabesity and other metabolic disorders. In a simple way, browning consists in the transformation of WAT into BAT. This adipose tissue is responsible for the process of thermogenesis and differs from the first one in its ability to regulate triglyceride levels and insulin sensitivity, mainly by the production of batokynes [238]. Although physical activity, or low temperatures are the principal factors regulating browning, it has also been described how the gut microbiota may be implicated in this process, thanks to its impact in bile acids metabolism, endotoxemia, the endocannabinoid system or through SCFA production [239,240]. Furthermore, Li et al. demonstrated how the intermittent fasting could stimulate this browning due to its modulatory properties on gut microbiota in murine models, although still more studies are required to prove the efficacy of this strategy [241].

Gut microbiota bacteria may also be used as biomarkers in patients with diabesity, although their validity is starting to be investigated. Wang et al. reported the utility of *Phocea*, *Pseudoflavonifractor* and *Lactobacillus intestinalis* as prognosis biomarkers in ZDF rats with diabesity, whose presence is associated with the worst metabolic serum profiles [242]. Dao et al. found that the grade of appearance of *Akkermansia muciniphila* could be used as a metabolic status biomarker, directly correlated with glucose homeostasis, serum lipid levels and fat redistribution in a dietary intervention in patients with obesity [243]. *Akkermansia muciniphila* equally represents a promising therapy in diabesity. One study conducted by Cani and De Vos showed how daily oral administration of 200 million of bacteria could revert the obesity induced by diet in mice [244]. These results were supported by a clinical trial reporting the benefits of targeting *A. muciniphila* in patients with diabesity and other metabolic conditions related to obesity (NCT02637115).

In conclusion, the gut microbiota is assumed to be a promising focus of study to fully understand the development, establishment, and pathophysiology of the main processes involved in diabesity and other metabolic disorders. As figured in Table 1, clinical applications focused on the gut microbiota and its derived products may have an important impact in diabesity. Increasing evidence targets gut microbiota in this condition, although further research in humans is still needed. The gut microbiota is modulated by a large number of factors, diet being one of its key regulators. Diabesity is a disease that is mostly preventable, whose origin may be related to unhealthy lifestyles. The gut microbiota could be the link and the consequence of the high impact of lifestyle interventions in patients with diabesity, hence helping to understand the relevance of an adequate lifestyle tin preventing this condition. Finally, the gut microbiota represents, indeed, a promising target to diagnose the disease at early stages, being correlated to patients’ status. It even allows the anticipation of the response that a patient will have by receiving a certain therapy, encouraging clinicians to select the perfect alternative for these patients.

## Figures and Tables

**Table 1:** The impact of the main interventions in the clinical management of diabesity on gut microbiota. Likewise, gut microbiota could represent a promising target in future therapies which are currently being investigated.

Intervention	Representative Examples	Gut Microbiota		Implications	References
		Increase	Decrease		
Lifestyles	Mediterranean diet (High in plants-based compounds as fiber and polyphenols, polyunsaturated fats)	*Lactobacillus* sp. *Bifidobacterium* sp. *Prevotella* sp. *A. muciniphila* *F. prausnitzii*	*Clostridium* sp.	Endotoxemia prevention Prebiotic effects Reduction in Firmicutes/Bacteroidetes ratio	[189,190]
Probiotics and Prebiotics	Fructans (Inulin and fructooligosaccharides) Probiotic formulas	*A. muciniphila* *Lactobacillus* sp. *Bifidobacterium* sp.	-	Antimicrobian effects Reinforcement of intestinal barrier Energy homeostasis Satiety regulation Body weight control SCFA production Immunomodulatory effects	[197] (NCT02637115)
Bariatricsurgery	Roux-en-Y gastric bypass (RYGB)	*Prevotella* *Veillonella* *Roseburia*	*Firmicutes* *Odoribacter* *Clostridium* *Methanogenic archaeas*	Bile acid regulation Succinate reduction Lower glycaemia	[209,215,217]
	Sleeve gastrectomy (SG)	*Gammaproteobacteria*			
		*Actinobacteria*			
Pharmacotherapy	Metformin	*Arkemansia muciniphila*	*Bacteroides fragilis*	Inhibition of FXR signaling	[219,220]
	Incretin-based triagonists and coagonists	-	-	Nutrients-gut microbiota-endocannabinoid system-enteroendocrine cells signaling	[225,226]
Future targets (Currently developing)	Faecal microbiota transplantation	-	-	Potential effects on obesity and metabolic syndrome (Correlated with receiver’s microbiome richness and donors metabolic status)	[230]
	Phage therapy	-	-	Regulate bacterial communities Immunomodulatory effects	[231,235]
	Biomarkers	*Phocea* *Pseudoflavonifractor* *Lactobacillus intestinalis*	*A. muciniphila*	Serum metabolites profile Glucose and lipid homeostasis, fat redistribution	[242,243]
				Potential therapeutic uses	[244] (NCT02637115)
	Browning	-	-	Bile acids, endotoxemia, endocannabinoid system and SCFA modulation	[239,240]
